# Carpaine Promotes Proliferation and Repair of H9c2 Cardiomyocytes after Oxidative Insults

**DOI:** 10.3390/ph15020230

**Published:** 2022-02-15

**Authors:** Suhaini Sudi, Yee-Zheng Chin, Nur Syafinaz Wasli, Siat-Yee Fong, Sadia Choudhury Shimmi, Siew-Eng How, Caroline Sunggip

**Affiliations:** 1 Department of Biomedical Sciences, Faculty of Medicine and Health Sciences, University Malaysia Sabah, Kota Kinabalu 88400, Sabah, Malaysia; suhainisudi@gmail.com (S.S.); nursyafinaz1992@gmail.com (N.S.W.); siatyee@ums.edu.my (S.-Y.F.); shimmi_cmc40@ums.edu.my (S.C.S.); 2 Department of Bioscience, Faculty of Applied Sciences, Tunku Abdul Rahman University College, Setapak 53300, Kuala Lumpur, Malaysia; briancyz-wl19@student.tarc.edu.my; 3 Borneo Medical and Health Research Centre, Faculty of Medicine and Health Sciences, Universiti Malaysia Sabah, Kota Kinabalu 88400, Sabah, Malaysia; 4 Faculty of Science and Natural Resources, Universiti Malaysia Sabah, Kota Kinabalu 88400, Sabah, Malaysia; sehow@ums.edu.my

**Keywords:** carpaine, *Carica papaya*, cardiomyocytes, proliferation, ischemia, reperfusion

## Abstract

Carpaine has long been identified as the major alkaloid in *Carica papaya* leaves that possess muscle relaxant properties. Limited study on the molecular signaling properties of carpaine urges us to conduct this study that aims to elucidate the mechanism underlying the cardioprotective effect of carpaine in embryonic cardiomyocytes of the H9c2 cell line. The 50% inhibitory concentration (IC_50_) of carpaine was first determined using a colorimetric MTT assay to establish the minimum inhibitory concentration for the subsequent test. Using a 1 µM carpaine treatment, a significant increase in the H9c2 proliferation rate was observed following 24 and 48 h of incubation. A Western blot analysis also revealed that carpaine promotes the upregulation of the cell cycle marker proteins cyclin D1 and PCNA. Carpaine-induced H9c2 cell proliferation is mediated by the activation of the FAK-ERK1/2 and FAK-AKT signaling pathways. In the setting of ischemia-reperfusion injury (IRI), carpaine provided a significant protective role to recover the wounded area affected by the hydrogen peroxide (H_2_O_2_) treatment. Furthermore, the oxidative-stress-induced reduction in mitochondrial membrane potential (MMP) and overproduction of reactive oxygen species (ROS) were attenuated by carpaine treatment. The current study revealed a novel therapeutic potential of carpaine in promoting in vitro cardiomyocyte proliferation and repair following injury.

## 1. Introduction

During embryonic and fetal heart development, cardiomyocytes undergo high-rate proliferation to form mature heart chambers to accommodate the high demand of the systemic circulation [1]. However, the proliferation rate gradually declines during the postnatal stage, where cardiomyocytes stop dividing and switch to hypertrophic growth when exposed to an increased workload [2]. Ischemia-reperfusion injury (IRI), a highly complex pathological event where the re-establishment of blood flow takes place following an ischemic injury can exacerbate cellular dysfunction and cardiomyocyte death [3]. Consequently, the recovery process of the adult heart after IRI is limited, due to the terminally differentiated and rarely dividing characteristic of the adult cardiomyocytes. This limitation makes it challenging to regenerate and replace the damaged and dead cardiomyocytes. Currently, there is no curative treatment to induce cardiomyocyte regeneration after injury.

For the past few decades, researchers have been attempting new therapeutic intervention strategies for heart repair following injury. Two major categories of the focused strategy are the transplantation of exogenous stem cells and stimulation of the proliferation of endogenous cardiomyocytes [4,5]. The emergence of induced pluripotent stem cell (iPSC)-derived cardiomyocytes has shown a promising therapeutic option for treating cardiovascular disease through transplantation. However, a limitation of the differentiation protocols resulted in the heterogeneity of the cardiac cell types, with varying proportions of ventricular-, atrial-, and nodal-like myocytes and non-myocytes [6,7]. Inevitably, this can lead to an increase in the risk of arrhythmogenesis and the formation of teratomas from the undifferentiated cells. The stimulation of an endogenous repair mechanism has become a novel approach in the modulation of cell cycle checkpoints to regain the proliferative capacity of adult cardiomyocytes. The activation of signaling cascades, such as the mitogen-activated protein kinase (MAPK) and phosphoinositide 3-kinase (PI3K)/protein kinase B (Akt) is known to facilitate the transformation of a post-mitotic cell into an actively dividing cell [8,9,10,11]. The introduction of therapeutic stimulation acts as a paracrine factor able to induce intracellular signaling pathways for cardiomyocyte proliferation. Insulin-like growth factor-1 (IGF-1) and fibroblast growth factor-1 (FGF-1) are among the secreted signaling proteins that activate PI3K-AKT signaling-mediated cardiomyocyte cell cycle entry [12,13].

Bioactive compounds derived from medicinal plants, such as alkaloids and flavonoids, serve as attractive stimulants for cardiovascular medicine in the context of cardiac regeneration following injury [14,15,16]. One of the interesting medicinal plants reported to contain such compounds is *Carica papaya* Linn. The leaves of the *C. papaya* tree have been reported to have diverse biological properties, such as anti-malarial, anti-inflammatory, anti-oxidant, and vasodilatory effects [17,18]. The phytochemical investigation suggested that *C. papaya* leaves possess a high content of alkaloids (carpaine) and flavonoids (quercetin and kaempferol) [17,19]. The cardioprotective mechanisms of quercetin and kaempferol have been elucidated previously following IRI. Quercetin protects embryonic H9c2cardiomyocytes from the IRI-induced hyperinflammatory response by the modulation of a signal transducer and the activator of transcription 3 (STAT3), while kaempferol exerts the same effect via the inhibition of mitochondria-mediated cardiomyocyte apoptosis [20,21]. For now, the involvement of carpaine in the mechanism of cardioprotection against IRI remains obscure.

Carpaine, a major type of alkaloid present in the *C. papaya* leaves, has long been known to markedly reduce blood pressure and heart rate, as well as exhibit spasmolytic activity for smooth muscle relaxation [22,23]. Several biological properties of carpaine have been reported, including anti-microbial and anti-plasmodial properties [17,24]. However, there is very limited literature currently available on the carpaine mechanism of action specifically in cardiovascular signaling. Therefore, in this study we aim to evaluate the potential benefits of carpaine as a therapeutic stimulator for cardiomyocyte proliferation and determine whether this in vitro proliferative activity is able to provide protection following IRI.

## 2. Results

### 2.1. Carpaine Promotes Proliferation in H9c2 Cells

**Figure 1:**
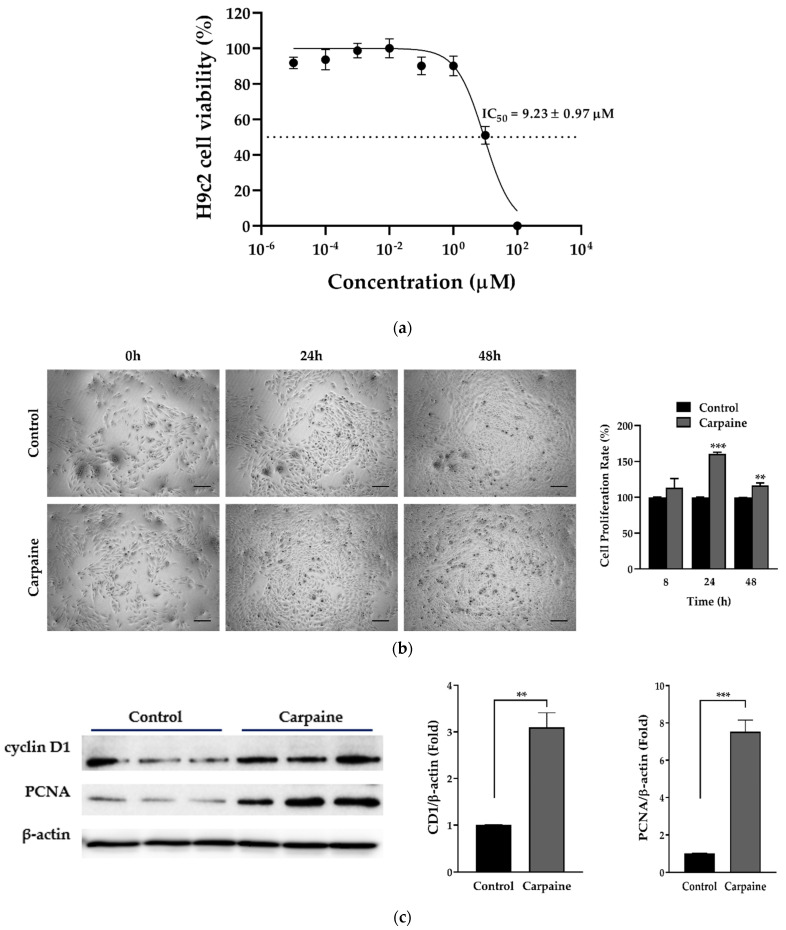
Carpaine promotes cell proliferation in H9c2 cells. (**a**) Cytotoxicity effect of carpaine on H9c2. Cells were treated with serial dilution of carpaine for 24 h to determine the 50% inhibitory concentration (IC_50_) (*n* = 6). (**b**) The effect of carpaine on the H9c2 proliferation rate. Representative images of H9c2 proliferation rate induced by carpaine treatment and quantitative analysis of cell proliferation. H9c2 treated with or without carpaine (1 µM) for 8, 24, and 48 h. Scale bar: 200 µm. (**c**) Carpaine promotes the activation of proliferation markers. H9c2 cells were treated with or without carpaine (1 µM) for 30 min (*n* = 3). Representative Western blot images of cell cycle markers and the quantification of cyclin D1 and proliferating cell nuclear antigen (PCNA) levels, normalized to β-actin. The protein band intensity is expressed as the fold change compared to control (non-treated group). Quantitative data are shown as the means ± SEM (*n* = 3). ** *p* < 0.01, *** *p* < 0.001, significantly different, as indicated by unpaired t-tests.

We investigated carpaine-induced cell proliferation using embryonic cardiomyocytes of the H9c2 cell line. We first determined the cytotoxic effect of carpaine on the H9c2 cells by using an MTT assay. The 50% inhibitory concentration (IC_50_) was recorded at 9.23 ± 0.97 µM (Figure 1a). Therefore, the minimum effective concentration was chosen based on 1/10 of the IC_50_ value, which is approximately 1 µM. We next demonstrated that treatment with 1 µM carpaine significantly increased the H9c2 cell proliferation rate after 24 (*p* < 0.001) and 48 h (*p* < 0.01) of incubation (Figure 1b). To confirm the effect of carpaine on H9c2 proliferation, we determined the involvement of the cell cycle marker proteins cyclin D1 and proliferation cell nuclear antigen (PCNA). Treatment with carpaine significantly increased cyclin D1 (*p <* 0.01) and PCNA (*p* < 0.001) protein levels as compared to the control non-treated group (Figure 1c). The increased level of proliferation markers suggests that carpaine promotes H9c2 cell proliferation.

### 2.2. Carpaine Triggers Upstream FAK-Induced ERK1/2 and AKT Activation in H9c2 Cells

**Figure 2:**
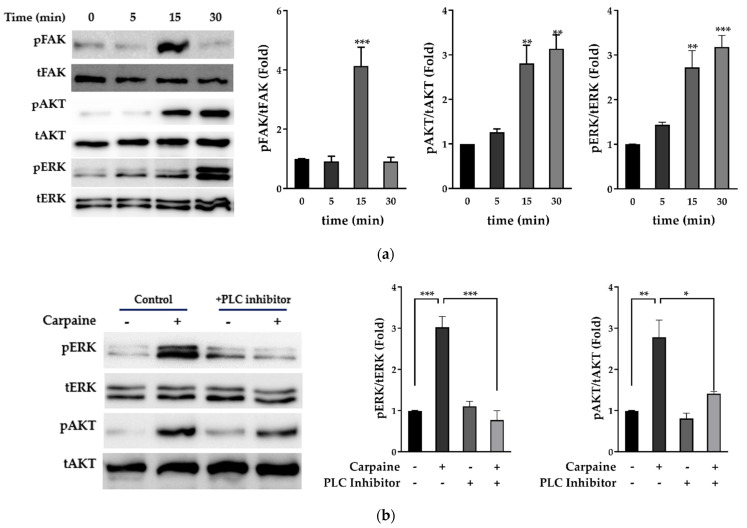
Carpaine triggers upstream FAK-induced ERK1/2 and AKT activation in H9c2 cells. (**a**) Carpaine treatment induces FAK, AKT, and ERK1/2 phosphorylation in H9c2 cells in a time-dependent manner. H9c2 treated with or without carpaine (1 µM) for 0, 5, 15, and 30 min. Representative Western blot images and the quantification of phosphorylated FAK (pFAK), phosphorylated AKT (pAKT), and phosphorylated ERK (pERK) levels, normalized to total FAK (tFAK), total AKT (tAKT), and total ERK (tERK), respectively. The protein band intensity is expressed as the fold change compared to the 0 min group. (**b**) Carpaine treatment mediates H9c2 proliferation via ERK1/2 and AKT activation. H9c2 cells were pre-treated with 1 µM PLC inhibitor (U73122) for 20 min followed by treatment with or without carpaine (1 µM) for 30 min. Representative Western blot images and the quantification of the pERK and pAKT levels, normalized to tERK and tAKT, respectively. The protein band intensity is expressed as the fold change compared to the non-treated control group. Quantitative data are shown as the means ± SEM (*n* = 3). * *p* < 0.05, ** *p* < 0.01, *** *p* < 0.001, significantly different, as indicated by a two-way ANOVA with Tukey’s comparison test.

Focal adhesion kinase (FAK) is highly activated during growth and development, which subsequently instigate diverse pathways in myocyte proliferation, such as mitogen-activated protein kinases (MAPKs) and protein kinase B (AKT) [25,26]. Therefore, we examined whether carpaine promotes proliferation via the activation of MAPKs, extracellular signal-regulated kinase (ERK), and AKT signaling pathways. Following various time points, the treatment with carpaine showed early activation of upstream FAK protein (15 min; *p* < 0.001) followed by the time-dependent activation of ERK1/2 (*p* < 0.001) and AKT (*p* < 0.01) which peaked at 30 min of incubation (Figure 2a). We further confirmed that carpaine induced cardiomyocyte proliferation via the ERK and AKT pathways by using the phospholipase C (PLC) inhibitor (U73122), which is involved in the activation of both ERK and AKT. Treatment with 1 µM U73122 significantly attenuated the phosphorylation of ERK1/2 (*p* < 0.001) and AKT (*p* < 0.05) as compared to the carpaine-treated group (Figure 2b). Thus, this finding suggests that carpaine promotes cardiomyocyte proliferation via the ERK1/2 and AKT signaling pathways following upstream FAK activation.

### 2.3. Carpaine Modulates the Wound-Healing Response by Stimulating Cardiomyocyte Proliferation in the Ischemic/Reperfusion Model

**Figure 3:**
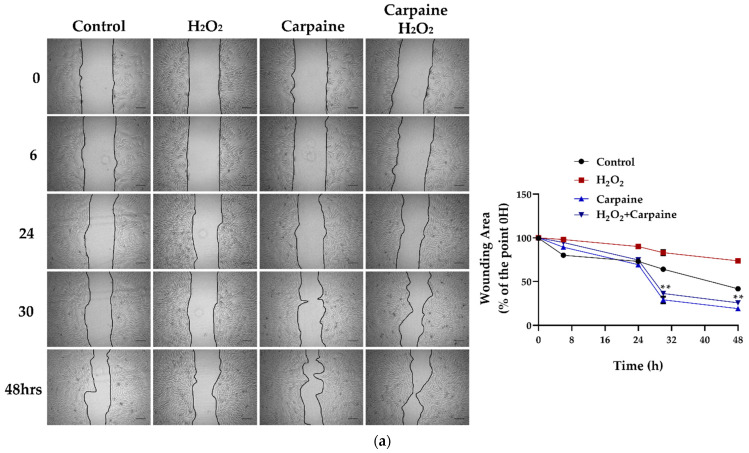

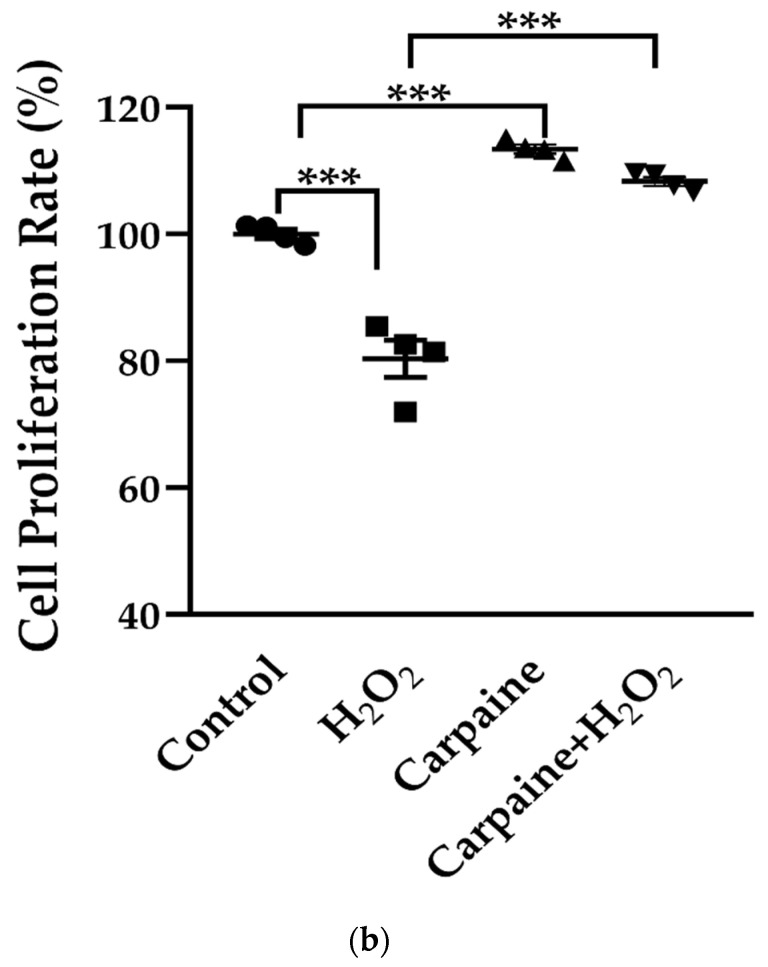
Carpaine modulates the wound-healing response by stimulating cardiomyocyte proliferation in an ischemic/reperfusion model. H9c2 were pre-treated with 1 µM carpaine for 1 h, followed by treatment with or without H_2_O_2_ (500 µM) for 1 h. (**a**) Representative wound healing images (**upper**) and the quantitative data of wounding area (**below**) were taken at different time points (0, 6, 24, 30, and 48 h). The data are represented as the percentage of time point 0 h of each group. Scale bar: 200 µm. (**b**) The cell proliferation rate of the H9c2 cells was determined using an MTT assay following treatment with or without carpaine and H_2_O_2_. The data are represented as percentages of the control. The data are shown as the means ± SEM (*n* = 3). ** *p* < 0.01, *** *p* < 0.001, significantly different, as indicated by a two-way ANOVA with Tukey’s comparison test.

Cardiomyocyte death and infarction are commonly associated with ischemic/reperfusion injury (IRI) [27]. Next, we assessed whether the proliferative effect of carpaine was effective following the IRI model in vitro. In this study, we incited a wound and IRI by scratching the cells, followed by incubation with hydrogen peroxide (H_2_O_2_) for 1 h, with or without carpaine pre-treatment. After 30 and 48 h of incubation, the wounded area of the H_2_O_2_-treated H9c2 cells was significantly larger compared to the carpaine pre-treatment with or without H_2_O_2_ (*p* < 0.01; Figure 3a). These results were supported by the MTT proliferation assay in which the pre-treatment with carpaine significantly increased the H9c2 proliferation rate following H_2_O_2_ incubation (*p* < 0.001; Figure 3b). These results indicate the protective role of carpaine by improving the wound healing ability through the proliferation of cardiomyocytes in response to IRI.

### 2.4. Carpaine Provides Protection against H_2_O_2_-Induced Oxidative Stress in H9c2 Cells

**Figure 4:**
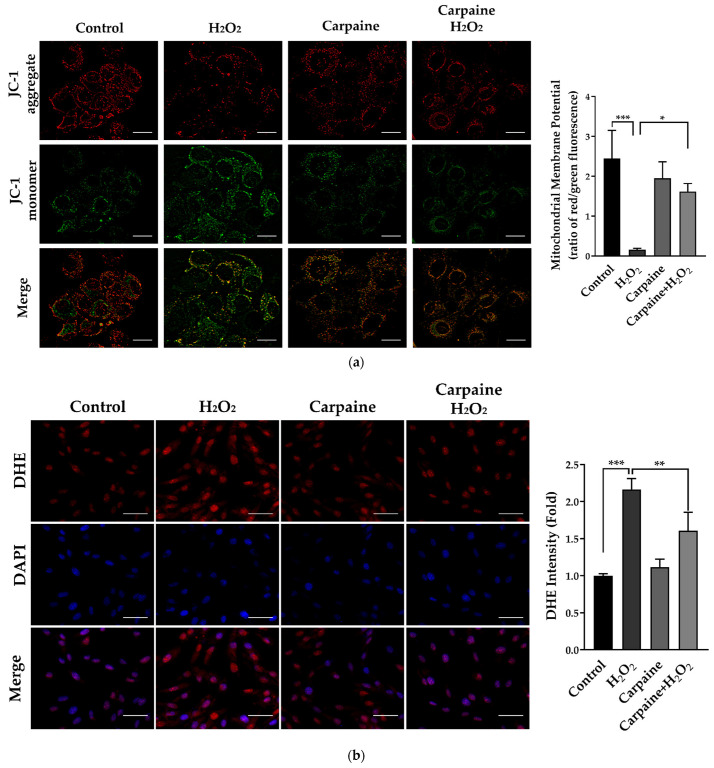
Carpaine provides protection against H_2_O_2_-induced IRI and oxidative stress in H9c2 cells. H9c2 were pre-treated with or without 1 µM carpaine for 1 h, followed by treatment with or without H_2_O_2_ (500 µM) for 1 h. (**a**) Representative mitochondrial membrane potential images (**upper**) and quantitative red to green fluorescent ratio (**lower**), determined using a JC-1 probe. Scale bar: 20 µm. (**b**) Representative dihydroethidium (DHE) fluorescence images (**left**) and semiquantitative results of the average DHE fluorescence intensity (**right**) in H9c2 cells. Scale bar: 50 µm. The data are shown as the means ± SEM (*n* = 3). * *p <* 0.05, ** *p* < 0.01, *** *p* < 0.001, significantly different, as indicated by a two-way ANOVA with Tukey’s comparison test.

Accumulated evidence has shown that H_2_O_2_ could result in the collapse of the mitochondrial membrane potential (MMP) [28]. We determined the MMP using a JC-1 probe in which fluorescent red represents healthy mitochondria and, in contrast, green represents unhealthy mitochondria. We demonstrated that treatment with carpaine attenuated the H_2_O_2_-induced decrease in the MMP in H9c2 cells. Carpaine significantly improved the recovery of the MMP following the oxidative stress caused by H*_2_*O*_2_* treatment (*p* < 0.05; Figure 4a). Myocardial IRI causes excess reactive oxygen species (ROS) production that affects intercellular connections and the cytoskeleton, eventually leading to cardiac dysfunction [20]. In this study, we used H_2_O_2_, which is known as the most stable ROS, to evaluate the protective effect of carpaine during oxidative stress in cardiomyocytes. Pre-treatment with carpaine significantly suppressed H_2_O_2_-induced oxidative stress in H9c2 cells as compared to the H_2_O_2_-treated group (*p* < 0.01; Figure 4b). These findings suggest the potential protective role of carpaine in the setting of oxidative stress in cardiomyocytes.

## 3. Discussion

**Figure 5:**
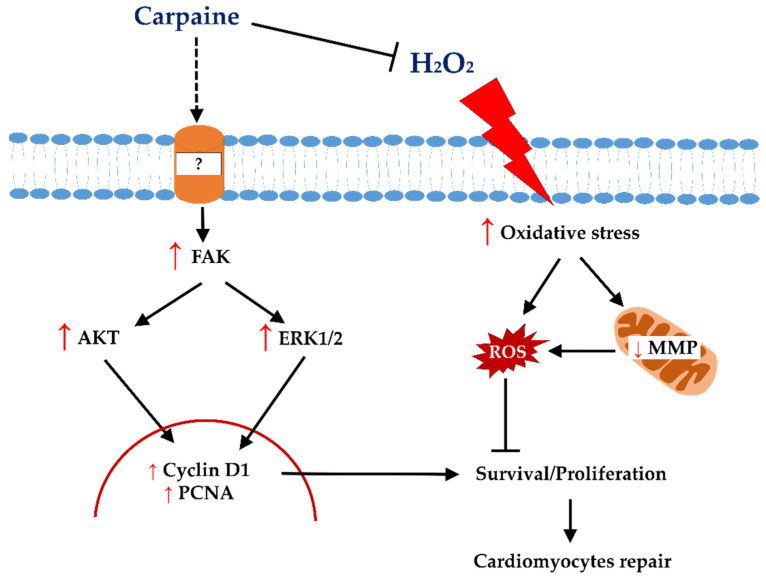
Proposed proliferative and protective signalling of carpaine in H9c2 cells. Carpaine induces cardiomyocyte proliferation via the FAK-ERK and FAK-AKT pathways and provides protection against H_2_O_2_-induced oxidative stress in H9c2 cells.

The stimulation of endogenous cardiomyocytes for cell cycle re-entry has been brought forward as novel approach to repair cardiac damage. Although cardiomyocytes undergo mitosis, failed cytokinesis marks the exit from cell cycle [29]. At this point, the transition of cardiomyocyte growth from hyperplasic to hypertrophy takes place immediately after birth [30]. However, emerging evidence have demonstrated that adult cardiomyocytes can ‘unlock’ their regenerative capacity by re-entering the cell cycle and produce new cardiomyocytes after ischemic injury [31,32]. Several studies have attempted to find the therapeutic potential of plant molecules that can alleviate the aggravated effect of post-ischemic/reperfusion injury (IRI). Although there is a very limited study of carpaine’s mechanism of action, a recent study on young *C. papaya* leaves was reported to contain highest amount of carpaine with anti-oxidant properties [33]. In addition, it has long been known that carpaine can regulate calcium influx, which is crucial during cardiomyocyte proliferation [22,23]. Based on this notion, we postulate that carpaine might possess protective and regenerative effects on cardiomyocytes. In this study, we revealed the underlying mechanism of the cardioprotective effect of carpaine, a major alkaloid contained in *C. papaya* leaves, in H9c2 cells (Figure 5).

A previous study showed that zebrafish and mice regain heart regeneration via the proliferation of pre-existing cardiomyocytes and not endogenous progenitor or stem cells [34,35]. Several possible mechanisms underlying cardiomyocyte proliferation have been extensively reviewed, including the modulation of cell cycle regulators and the activation of the MAPK and PI3K-AKT signaling pathways [36,37]. A positive cell cycle regulatory effect of AKT is reported to prolong the activation of the cell cycle regulator cyclin D1 (CD1) in hepatocellular carcinoma [38]. In neonatal cardiomyocytes, activation of AKT augmented cell-cycle-dependent kinases for S and G2/M phase transitions, thereby leading to cell division [39]. A more recent study demonstrated that AKT-dependent cyclin D1 activation promotes cardiomyocyte proliferation in response to microRNA treatment [9]. In addition, proliferating cell nuclear antigen (PCNA) is commonly used to identify DNA synthesis, as well as DNA repair, by forming a complex with CD1 [40]. In this study, we demonstrated that carpaine significantly promotes proliferation via increased levels of CD1 and PCNA, presumably via Akt pathway activation (Figure 1 and Figure 2). Therefore, the activation of the AKT pathways by carpaine led to a high rate of H9c2 proliferation as compared to the control group (Figure 2a).

The involvement of MAPK has been widely reported in cardiomyocyte proliferation. MAPK p38 negatively regulates cardiomyocyte division by inducing cell cycle exit and differentiation both in vitro and in vivo [41,42]. In contrast, activation of MAPK ERK1/2 via the Raf-MEK-ERK cascade positively regulates cardiomyocyte proliferation as a downstream target of growth factors such as fibronectin and collagen [11,43]. In another study, it was found that activation of ERK1/2 via the neuregulin-1-activated receptor tyrosine-protein kinase erbB-2 axis is beneficial to re-activate postnatal cardiomyocytes’ proliferative and regenerative ability [44]. The present study demonstrated that a high level of ERK1/2 activation in response to carpaine treatment is likely to contribute to the proliferative effect of this alkaloid (Figure 2a). To further confirm the involvement of the AKT and ERK1/2 proteins, significant suppression of the phosphorylation of both proteins was observed in response to upstream phospholipase C (PLC) inhibition by U73122, indicating the involvement of these pathways in carpaine-induced H9c2 proliferation (Figure 2b).

We further examined the possible upstream protein involved in the carpaine-induced H9c2 proliferation mechanism. Early activation of focal adhesion kinase (FAK) is observed in the carpaine-treated group (Figure 2a). Importantly, cardiac FAK’s protective role in IRI-induced cardiomyocyte apoptosis has been previously reported [45]. Our finding is supported by a previous study that reported basal activation of FAK in embryonic hearts is crucial to suppress MAPK p38 activity and allow cardiomyocyte cell cycle progression through G2/M phase [25]. The same study also showed that at the peak of myocyte proliferation, ERK1/2 is downregulated in FAK-null heart mice, indicating FAK-dependent ERK activation in the cell cycle progression. Additionally, FAK was shown to mediate the protective effect of artesunate in IRI-induced myocardial necrosis via the PI3K/AKT signaling pathway [46]. In the present study, we postulate that carpaine functions as a bridge to modulate FAK-dependent signals via AKT and ERK1/2 activations, promoting cardiomyocyte survival and proliferation (Figure 1 and Figure 2).

On the basis of the proliferative capability of carpaine in H9c2, we then evaluated the protective effect of this alkaloid in response to hydrogen peroxidase (H_2_O_2_)-induced IRI and oxidative stress. The present study showed that H_2_O_2_ causes a delay in wound healing and, in contrast, treatment with carpaine promotes a high proliferation rate of H9c2, which eventually closed the wound area faster than the H_2_O_2_ non-treated group (Figure 3). The reperfusion injury salvage kinase (RISK) pathway has been described as a group of protein kinases, such as PI3K/AKT and ERK1/2, that provide powerful protection at the time of myocardial reperfusion [47]. Therefore, we speculate that carpaine-induced AKT and ERK activation may provide cardioprotective effect against IRI by triggering the activation of survival signaling pathways.

Following IRI, excessive production of ROS can lead to cardiac dysfunction, which is the most acute cardiovascular disease [48]. The mitochondrial generation of superoxide and/or H_2_O_2_ are the major sources of ROS in IRI [49]. A prolonged period of ischemia mediates the opening of the mitochondrial permeability transition pore (mPTP) that occurs soon after reperfusion. A robust H_2_O_2_ level is reported following mPTP opening in ischemic/reperfused rat hearts [50]. In the present study, we demonstrated that the H_2_O_2_-induced mPTP opening shown by the JC-1 staining is attenuated in response to carpaine treatment (Figure 4a). Moreover, the excessive production of ROS by H_2_O_2_ was significantly suppressed in the carpaine-treated group (Figure 4b). ROS-induced ROS release (RIRR) is a positive feedback mechanism that leads to mitochondrial and cellular damage in IRI. The impact of oxidative stress causes accumulation of mitochondrial ROS exceeding the threshold, and that inevitably triggers the opening of mPTP. This leads to the collapse of the MMP, induces ROS release from mitochondria to the cytosol, and activates RIRR in neighbouring mitochondria [51]. In this study, we speculate that carpaine reduces H_2_O_2_- induced ROS overproduction through the inhibition of RIRR (Figure 4 and Figure 5).

In conclusion, the results of the present study indicate that carpaine positively regulates cardiomyocyte repair by increasing the proliferation rate via the FAK/AKT and FAK/ERK1/2-dependent pathways (Figure 5). This is the first report that revealed the underlying mechanism of the cardioprotective effect of carpaine. However, we are yet to provide direct evidence of the carpaine-regulated calcium influx in the setting of IRI-induced ROS overproduction. Therefore, future studies incorporating calcium and ROS interplay in the carpaine cardioprotective mechanism will be further verified.

## 4. Materials and Methods

### 4.1. Cell Line, Cell Culture, and Cell Treatment

Embryonic cardiomyocytes of the H9c2 cell line were obtained from Elabscience (Wuhan, China). H9c2 cells were cultured in Dulbecco’s modified Eagle medium (DMEM) (Nacalai Tesque) supplemented with 10% fetal bovine serum (FBS) and 1% penicillin/streptomycin in a humidified atmosphere of 95% air and 5% CO_2_ at 37 °C. The cell culture was treated with the phospholipase inhibitor U73122 (TargetMol, Boston, MA, USA) and carpaine purchased from BOC Sciences (New York, NY, USA), with 99.7% purity, tested using high-performance liquid chromatography. Oxidative stress and IRI were induced with media consisting of 500 µM hydrogen peroxide (H_2_O_2_) (Baker, Johnstown, PA, USA) for 1 h.

### 4.2. Cytotoxicity and Proliferation Assay

The cytotoxicity and proliferative effect of H9c2 cells were determined colorimetrically using a 3-(4,5-dimethylthiazol-2-yl)-2,5-diphenyltetrazolium bromide (MTT) assay. H9c2 cells were seeded at 2 × 10^4^ cells/well in 96-well plates. For the cytotoxicity assay, H9c2 cells were treated with the serial dilution of carpaine ranging from 10^−4^–10^2^ µM for 24 h to determine the 50% inhibitory concentration (IC_50_) of carpaine. For the proliferation assay, cells were treated with carpaine for 8, 24, and 48 h, and images were taken at each time point using an Olympus CKX41 inverted microscope. Following the incubation period, a 0.5 mg/mL final concentration of MTT solution was added to each well and incubated for 1–4 h at 37 °C. The supernatants from each well were removed, and precipitated formazan crystals were dissolved in 100 µL of DMSO. The absorbance was read at 560 nm using a GloMax Explorer Multimode plate reader (Promega).

### 4.3. Immunoblot Analysis

Carpaine or PLC inhibitor-treated H9c2 cells were harvested in an extraction buffer (Nacalai Tesque) consisting of 50 mM Tris-HCl (pH 7.6), 150 mM NaCl, 1% Nonidet P40 substitute, 0.1% SDS, 0.5% sodium deoxycholate supplemented with protease inhibitor cocktail (1:100 dilution factor), followed by scratching the cells on ice for sample collection. The mixture was then centrifuged at 10,000 rpm for 10 min at 4 °C. The total protein quantification was carried out using a DS-11 spectrophotometer (Denovix) with bovine serine albumin (BSA) as a standard. The cell lysates (15 mg/mL proteins) were then diluted with a 3:1 (*v*/*v*) sample buffer consisting of glycerol; 0.5 M Tris-HCl, pH 6.8; 10% SDS; 0.5% bromophenol blue; and β-mercaptoethanol for protein separation by sodium dodecyl sulphate-polyacrylamide gel electrophoresis (SDS-PAGE). The separated proteins were subjected to a semi-dry electro-transfer onto polyvinylidene difluoride (PVDF) membranes using a CAPS buffer system (0.1 M CAPS pH 10.5 and 20% *v/v* methanol) and blocked with 1% BSA diluted in Tris-buffered saline and 0.1% Tween 20 (TBST) for 1 h on a shaker. The membranes were then probed with specific primary monoclonal antibodies against FAK (1:1000; #3285), phosphor-FAK (1:1000; #3283), ERK1/2 (1:1000; #4695), phospho-ERK1/2 (1:1000; #4370), AKT (1:1000; #4691), and phosphor-AKT (1:1000; #4060) from Cell Signalling, polyclonal antibodies against cyclin D1 (1:1000; GTX108624) and PCNA (1:1000; GTX100539) from GeneTex and beta-actin (Elabscience, 20058, Texas, USA) as a loading control overnight at 4 °C. Following primary antibody incubation, the membranes were washed and incubated with IgG-HRP-conjugated specific secondary antibodies (anti-rabbit; #7074) from Cell Signaling Technology for 1 h on a shaker. The immunoreactive proteins were detected using Western Lightning Plus ECL (Perkin Elmer, Waltham, MA, USA), and images were captured with an ImageQuant LAS 4000 (GE Healthcare Life Science, Piscataway, NJ, USA). The band intensity was measured using ImageJ software (National Institutes of Health, Bethesda, MD, USA) and normalized with respective total protein band intensities.

### 4.4. Wound Healing Assay

The wound healing assay was conducted as previously described [20]. H9c2 cells were seeded at 1 × 10^5^ cells/well for 24 h to allow cell attachment. Prior to a 1 h pre-treatment with carpaine and a 1 h treatment with 500 µM H_2_O_2_, H9c2 cells were scraped with 10 µL tips to establish a wound area. Wound area images were captured at several time points (0, 6, 24, 30, and 48 h) and the percentage of wound healing was determined by comparing the images with the wound area at the 0 h time point.

### 4.5. Assessment of the Mitochondrial Membrane Potential (MMP)

To measure the effect of carpaine on H_2_O_2_-induced MMP in H9c2, 5,5,6,6′-tetrachloro-1,1′,3,3′ tetraethylbenzimi-dazoylcarbocyanine iodide (JC-1) dye was used to measure the state of mitochondrial polarization. JC-1 dye staining was conducted according to manufacturer instruction (E-CK-A301, Elabscience, Texas, USA). Briefly, following the incubation treatment, the culture medium was discarded, and the cells were washed with JC-1 buffer. JC-1 working solution was then added and the cells were incubated in a CO_2_ incubator for 20 min. The cells were then washed with JC-1 buffer and culture medium was added. Immediately, the fluorescence was observed using a BX63 automated upright microscope (Olympus, Tokyo, Japan) and the relative ratio of red and green fluorescence was measured and analyzed using ImageJ software to represent the mitochondrial depolarization.

### 4.6. ROS Production Analysis

To measure ROS production after treatment, 2 µM dihydroethidium (DHE) was added directly into treated and non-treated H9c2 cell cultures and incubated in a CO_2_ incubator for 1 h. The cells were washed twice with PBS and fixed in 2% PFA for 10 min. The cells were then washed with PBS and mounted on a glass slide with ProLong Gold Antifade Mountant with DAPI. The averaged DHE fluorescence intensity was observed using a BX63 automated upright microscope (Olympus), analyzed using ImageJ (National Institutes of Health) software, and quantified by the averaging intensity of 40–50 cells /field in each experiment. Changes were calculated by averaging the DHE intensity in each group compared with the non-treated control group.

### 4.7. Statistical Analysis

All data were presented as the means ± SEM from at least three independent experiments. Statistical comparisons for two groups were made using the unpaired *t* test and multiple groups were compared using a one-way ANOVA followed by Tukey’s *post hoc* test. A *p* value of <0.05 was set as the significance limit.

## Data Availability

The data presented in this study are available in this article.
